# Heat Stroke Leading to a Fatal Outcome

**DOI:** 10.7759/cureus.33226

**Published:** 2023-01-01

**Authors:** Som N Chalise, Ehsun Mirza, Raza Malik

**Affiliations:** 1 Pulmonary and Critical Care, Riverside Health System, Newport News, USA; 2 Gastroenterology and Hepatology, Albany Medical Center, Albany, USA

**Keywords:** multi-organ failure, acute encephalopathy, disseminated intravascular coagulation (dic), acute kidney injury, acute liver failure, heat stroke

## Abstract

Heat stroke (HS) can cause several physiological changes in the body. In its most severe form, it can cause multi-organ failure including encephalopathy, circulatory shock, liver failure, renal failure, disseminated intravascular coagulation, and rhabdomyolysis among others. HS is a preventable condition; however, it can be life-threatening in severe forms. We present a case of HS in a 54-year-old male, with rapidly progressive multi-organ failure and a fatal outcome along with a brief literature review.

## Introduction

Heat stroke (HS) is a preventable but potentially life-threatening condition. HS is characterized by an elevated body temperature of more than 40 degrees Celsius accompanied by hot, dry skin and central nervous system dysfunction (delirium, convulsion, or coma) [1]. HS can be non-exertional (classic) and exertional. HS usually responds to supportive care; however, it can lead to multiple complications including rhabdomyolysis, liver failure, acute kidney failure, disseminated intravascular coagulation, and ultimately death. We report a case of rapidly developed multi-organ failure resulting in death in an unfortunate 54-year-old patient due to heat stroke.

## Case presentation

A 54-year-old male patient with a history of seizures on phenytoin at home was brought to the emergency room (ER) for acute encephalopathy. Prior to the presentation, he worked outside mowing grass with a lawnmower on a hot summer day. After that, he rode his bike and fell off it without a witnessed accident or seizure activity. When paramedics arrived, he was unresponsive with a temperature of 108 degrees Fahrenheit (*F). He was then transported to the ER for further evaluation. An initial evaluation in ER revealed an unresponsive patient with a Glasgow Coma Scale score of 3, temperature of 107.2 *F, tachycardia with a heart rate of 163 beats per minute, respiratory rate of 36, blood pressure of 112/56 mm Hg, and oxygen saturation of 91% on 2 liters nasal cannula. Other systemic examinations were normal. He was promptly intubated for airway protection. Cooling maneuver was started in ER with ice packs in axilla/groin, wet gauze sheets on exposed body parts, and arctic-sun device with subsequent lowering of temperature to 101*F. During that time, his blood pressure dropped with mean arterial pressure in the 50s. He was given 30 cc/kg body weight crystalloid fluid bolus intravenously (IV), cultures were drawn, and started on empiric vancomycin and piperacillin-tazobactam. After that blood pressure stabilized to over 90 mmHg systolic and heart rate dropped down to 90-100. His temperature remained less than 102*F after the initial reduction in ER. CT scans of the head, cervical spine, chest, and abdomen/pelvis were unremarkable. Initial ER labs were also unremarkable including normal coagulation profile and liver functions (Table 1). He was then transferred to the medical intensive care unit for further management.

**Table 1:** Pertinent lab values summary WBC= White Blood cell; Hb= Hemoglobin; platelets in k/mcL, PT=Prothrombin time; INR=International normalized ratio; aPTT=Activated Partial Thromboplastin Time; BUN=Blood Urea nitrogen; AST=Aspartate Aminotransferase; ALT=Alanine aminotransferase; ALP=Alkaline Phosphatase; T.Bi.=Total Bilirubin; LDH=Lactate Dehydrogenase; CK=Creatine Kinase; TSH=Thyroid Stimulating Hormone; fT4= Free T4; fT3=Free T3; COVID-19= Coronavirus Disease 2019

	Initial labs	11 hrs	18 hrs	36 hrs	39 hrs	43 hrs	48 hrs	Reference values
WBC (k/mcL)	3.4	7.7	10	8.2	8.2	9.6	10.1	4.8-10.8
Eosinophils (k/mcL)	0.1							0.2-0.6
Hb (gm/dL)	13.3	13	12.8	11.2	11.3	10.7	10.3	14-18
Platelets	199	63	47	24	15	14	14	150-450
PT (seconds)	11.4		19.6	49.2		34.5	49.8	10-12.6
INR	1.04		1.87	5.11		3.48	5.19	0.9-1.1
aPTT (seconds)	21.2		28.1	59.9		50.4	52.2	20-35
Fibronogen (mg/dL)			199	70		183	142	200-400
Sodium (mmol/L)	143	144		143		142	142	135-148
Potassium (mmol/L)	3.5	3.0		3.9		4.5	4.6	3.5-5.5
Bicarb (mmol/L)	21	19		19		18	17	22-32
BUN (mg/dL)	9	19		26		31	31	7-24
Creatinine (mg/dL)	1.61	1.66		2.89		4.60	4.80	0.61-1.24
AST (U/L)	21	162		7145	7954	8265	8769	7-37
ALT (IU/L)	12	115		4167	4961	5437	6085	7-41
ALP (U/L)	90	87		132	144	147	187	32-129
T. Bi. (mg/dL)	0.5	0.9		2.6	3.2	3.8	4.6	0.1-1.5
LDH (U/L)					10,044			98-220
CK (U/L)	284	766		5853		7053		49-397
Lactic acid (mmol/L)	3.72	1.7						
Amylase (U/L)				230				28-100
Lipase (U/L)				324				10-60
Procalcitonin (ng/mL)	<0.05							<0.05
Troponin I (ng/mL)	0.00							0.00-0.08
TSH (uIU/mL)		0.43						0.34-3.7
fT4 (ng/mL)		2.8						0.58-1.64
fT3 (pg/mL)		1.22						2.0-3.9
Acetaminophen (mcg/mL)	2.9							10-25
Salicylate (mg/dL)	<1.5							15-30
Ethanol (mg/dL)	<10							
Urine toxicology screen	Negative							
Urinalysis	Negative for infection							
Blood and urine culture	No growth							
COVID-19, flu A/B	Negative							

Electroencephalogram monitoring did not show seizure activity but, however, showed a diffuse cortical slowing pattern. Supportive care was provided with maintenance IV crystalloid infusion, ventilator support, nutritional support, and close monitoring. Electrocardiogram was normal. Echocardiogram performed during intubated status showed a normal ejection fraction with a mildly dilated right ventricle chamber and an estimated right ventricular systolic pressure of 45 mmHg. Within the next 12 hours or so, his mental status slowly improved, and he started to follow commands intermittently. He was on minimal ventilator support and was hemodynamically stable. Despite improvement in his temperature, hemodynamics, and neurological status, he started to get worse in terms of rapid worsening of liver enzymes, worsening kidney function, rhabdomyolysis, thrombocytopenia, and coagulopathy. The details of lab values are given in Table 1. He had no signs of bleeding. He was given cryoprecipitate for a low fibrinogen level of 70 and was given two units of fresh frozen plasma to correct coagulopathy. He also developed hypoglycemia needing multiple doses of dextrose (the lowest glucose was 44 mg/dl). A diagnosis of heat shock was made as the etiology of his acute liver failure, acute kidney failure, and coagulopathy. His antinuclear antigen, anti-smooth muscle antibody, anti-liver kidney microsomal antibody, and acute hepatitis panel laboratory results were negative. He was then transferred to a tertiary center with capability for liver transplant. His clinical condition worsened at the tertiary center with worsening liver failure, acute kidney failure, and coagulopathy leading to a goals-of-care discussion, from which his management was directed towards comfort care only.

## Discussion

Classic (non-exertional) HS is usually seen in children or in elderly, chronically ill, and sedentary individuals, and medications may often remain on board. Exertional HS is usually seen in active individuals, due to excessive heat production more than heat loss, during strenuous physical activity [2]. Drug abuse like cocaine, amphetamine, or synthetic stimulants also contributes to exertional HS. Young athletes, outdoor workers, and military personnel are the demographics at risk of exertional HS. Our patient had exertional HS due to outdoor physical work during a hot summer day followed by riding a manual bicycle outdoors. We do not know the status of his hydration on that day. He did not have any additional medicines including alcohol, cocaine, methamphetamine, etc. based on his history and based on our tests. Phenytoin has been described as a causative drug for high fever, particularly in association with rash, lymphadenopathy, and elevated liver enzymes in hypersensitivity reaction [3], however, in our case it is less likely since he was on phenytoin for over two decades without any issues and there were no other signs. Drug reaction with eosinophilia and systemic symptoms was also unlikely due to the absence of eosinophilia and timing (it usually starts two to eight weeks after the offending drug) [4].

Climate change with global warming and associated deaths has made the topic of heat stroke more apparent and several articles have been published in the last decade in this regard. These are potentially preventable deaths.

When our body temperature increases, the normal response is vasoconstriction at core vessels and vasodilation of peripheral vessels to allow heat dissipation. An increase in cardiac output and minute ventilation also helps in heat transmission. Heat exchange is dependent upon the gradient between the individual and the surrounding temperature and humidity. Hence, HS affects individuals more often during hot and humid weather [5]. There is decreased perfusion to the core organs including the liver, kidney, and splanchnic circulation resulting in possible ischemic injury to these organs. Cytokines are released from ischemic organs and endothelium. Other proposed mechanisms include direct injury to the liver and endothelium due to excessive heat, micro-thrombosis, or endotoxemia [6]. HS-mediated inflammatory response shows a similar effect to systemic inflammatory response syndrome [1, 7]. HS was the likely factor in our patient's profound and rapid liver failure, kidney failure, and disseminated intravascular coagulation (DIC) (manifested as thrombocytopenia, coagulopathy, and low fibrinogen). It is important to note that initial laboratory data did not show organ dysfunctions. However, they started to get rapidly worse after 36 hours or so; there may be delayed effects of heat stroke on the body in some patients for unclear reasons. There was a brief hypotensive episode due to heat stroke initially, which was treated with IV fluid resuscitation in ER. Brief hypotension by itself is unlikely to cause such a profound organ dysfunction seen in this patient. 

It is very important for prompt diagnosis and initiation of treatment since delayed treatment has higher mortality. Differential diagnoses in a case of extremely high temperatures and neurological dysfunction are important to consider and may include certain infections (meningitis, encephalitis), drug intoxications (cocaine, amphetamine, atropine), severe dehydration and metabolic syndromes (neuroleptic malignant syndrome, serotonin syndrome, malignant hyperthermia, etc) [2]. Treatment is mostly supportive and involves cooling the body temperature rapidly [2,8]. Core temperature is to be measured with a rectal probe and there must be prompt cooling to less than 39 degrees Celsius [2]. Rapid cooling can be achieved with immersion into cold water, pouring water on the body and fanning, infusion of cold IV fluids, application of ice packs, or wet gauze sheets and fanning [2]. Medications used to lower body temperature like acetaminophen and aspirin become ineffective in heat stroke and may be deleterious causing liver damage and bleeding complications. Supportive care for organ damage is recommended. Some cases of liver failure may need liver transplant, the criteria of which have not been well studied in the case of HS.

## Conclusions

HS is a rare condition, especially where there is access to air-conditioning indoors. Usually, it responds to supportive care. However, it can be progressive leading to multi-organ failure, and can potentially be fatal. We should begin supportive care immediately and monitor the patient for potential complications. It is important to recognize the complications early on as they can progress very rapidly.
